# Adsorption and Release of Sulfamethizole from Mesoporous Silica Nanoparticles Functionalised with Triethylenetetramine

**DOI:** 10.3390/ijms22147665

**Published:** 2021-07-17

**Authors:** Cristina Carucci, Nicola Scalas, Andrea Porcheddu, Marco Piludu, Maura Monduzzi, Andrea Salis

**Affiliations:** 1 Dipartimento di Scienze Chimiche e Geologiche, Università di Cagliari, Cittadella Universitaria, SS 554 Bivio Sestu, 09042 Monserrato, CA, Italy; cristina.carucci@unica.it (C.C.); nicola.scalas@tiscali.it (N.S.); porcheddu@unica.it (A.P.); monduzzi@unica.it (M.M.); 2 Consorzio Interuniversitario per lo Sviluppo dei Sistemi a Grande Interfase (CSGI), via Della Lastruccia 3, 50019 Sesto Fiorentino, FI, Italy; mpiludu@unica.it; 3 Dipartimento di Scienze Biomediche, Università di Cagliari, Cittadella Universitaria, SS 554 Bivio Sestu, 09042 Monserrato, CA, Italy

**Keywords:** MSN, antimicrobial drug, adsorption, kinetics, isotherms, release

## Abstract

Mesoporous silica nanoparticles (MSN) were synthesised and functionalised with triethylenetetramine (MSN-TETA). The samples were fully characterised (transmission electron microscopy, small angle X-ray scattering, Fourier transform infrared spectroscopy, thermogravimetric analysis, zeta potential and nitrogen adsorption/desorption isotherms) and used as carriers for the adsorption of the antimicrobial drug sulphamethizole (SMZ). SMZ loading, quantified by UV–Vis spectroscopy, was higher on MSN-TETA (345.8 mg g^−1^) compared with bare MSN (215.4 mg g^−1^) even in the presence of a lower surface area (671 vs. 942 m^2^ g^−1^). The kinetics of SMZ adsorption on MSN and MSN-TETA followed a pseudo-second-order model. The adsorption isotherm is described better by a Langmuir model rather than a Temkin or Freundlich model. Release kinetics showed a burst release of SMZ from bare MSN samples (k_1_ = 136 h^−1^) in contrast to a slower release found with MSN-TETA (k_1_ = 3.04 h^−1^), suggesting attractive intermolecular interactions slow down SMZ release from MSN-TETA. In summary, the MSN surface area did not influence SMZ adsorption and release. On the contrary, the design of an effective drug delivery system must consider the intermolecular interactions between the adsorbent and the adsorbate.

## 1. Introduction

The goal of an efficient drug delivery system (DDS) is to directly transport the correct amount of therapeutic molecules to the target with a tailored and controlled release [1]. Among nanocarriers for in situ drug delivery, mesoporous silica nanoparticles (MSNs) are likely one of the most promising [2,3]. MSNs have a regular highly reproducible, ordered, mesoporous structure constituted by cylindrical pores (pore size 2–5 nm), with a high surface area (~700–1000 m^2^ g^−1^) that provides high drug loadings [4]. MSN surface functionalisation plays more than one important role, increasing their biocompatibility [5], inserting targeting molecules [6] as well as hosting stimuli-responsive moieties [7,8]. Among various types of MSN, MCM-41 (Mobile Composition of Matter No. 41) has been widely studied in biomedicine [9], thanks to their ordered parallel pore channels that facilitate drug diffusion [10,11,12,13]. For all these reasons, many studies have explored the use of functionalised MSNs (with MCM-41 matrix) as drug delivery systems for a wide range of applications such as anticancer [14], antiviral [15] antituberculosis [16], anti-inflammatory [17], antihypertensive [18], and antibacterial treatments [19,20,21,22,23,24,25,26]. For example, Lu et al. reported high tumour suppression effect in vivo of camptotecin loaded on MSN for cancer cells target [27]. To develop an antiviral DDS, Lee and co-workers modified MSNs with glycosaminoglycan to inhibit the viral entry of herpes simplex virus [28]. In vitro release of Rifampim, an anti-tuberculosis drug, was tested from bare MSN achieving up to 95% drug release in 24 h [16]. In another work, Gounani et al. loaded polymyxin B on MSNs obtaining a high antibacterial efficiency and an improved biocompatibility of the DDS [29].

Nowadays, antibiotic resistance is one of the biggest health threats [30]. Infections caused by bacteria resistant to common antibiotics have become a global healthcare problem in the 21st century. By 2050, the development of antibiotic-resistant bacteria will cause severe infections difficult to diagnose and treat [31,32]. Common health treatments are at risk due to superbugs (multi-drug resistant bacteria) that persist in hospitals (i.e., methicillin-resistant *Staphylococcus aureus*) [33].

Among other antimicrobial drugs of high and very high importance such as glycopeptides, lipopeptides, penicillins, quinolones [34], sulphonamides have been studied as bacteriostatic drugs, particularly against the hard-to-treat resistant bacteria resulting in nosocomial infections [35]. Sulphonamides inhibit the enzymatic synthesis of folic acid by competing with the natural substrate p-aminobenzoic acid of dihydropteroate synthase, thus reducing the effective enzyme activity [36]. Folic acid is an essential nutrient necessary for protein and nucleic acid synthesis (DNA and RNA) in bacteria cells. As a result of folic acid biosynthesis inhibition, bacterial cells stop growing, preventing their reproduction and infectivity [37,38]. Sulphonamide drugs are also used as topical ointment for burn wounds [39]. Among them sulphamethizole (SMZ) is a drug used in the treatment of urinary tract infections [40].

Several research papers investigated the drug delivery of sulphonamides. Ghedini et al. studied a DDS for the controlled release of silver sulphadiazine for topical administration from hybrid gels using chitosan and silica as precursors [41]. Recently, Rosas et al. studied the release of the same compound from MIL-53(Al) MOFs reaching up to 80% release in physiological conditions [42]. Suman et al. reported the impregnation of silver sulphadiazine on SBA-15 mesoporous silica reaching a 95% release in simulated body fluid after 48 h [43].

According to Vallet-Regì and co-workers, tailored functionalisation of silica nanoparticles is fundamental in developing the third generation DDS [21,44,45,46]. Owing to the strong presence of peptidoglycan and phosphate groups, most bacterial cell walls are negatively charged. Hence, positively charged nanoparticles may interact with bacteria cell walls through electrostatic forces. Casey et al. analysed the bacterial viability with three different types of functionalised MSNs, amine (positive), carboxylic (negative) and aromatic (neutral) finding that the positively charged functionalisation resulted in a reduction of *Staphylococcus aureus* biofilms [47]. Similarly, Zaharudin et al. analysed the loading and release of quercetin and gemcitabine from functionalised MSN-NH_2_, MSN-SH and MSN-COOH [48]. Through the same antibacterial mechanism, Niu et al. coated silver core MSN with a cationic polymer QPEI (quaternary ammonium polyethyleneimine). Owing to the high positive charge of the so-constructed Ag@MSN-QPEI, the DDS showed antibacterial effect against Gram negative *Pseudomonas syringae* [49]. Dendrimers of third generation (G3), composed of high concentration of positively charged amino groups, were also used as external MSN functionalisation resulting in exceptional disruption of *E.Coli* biofilm [19]. Mas et al. decorated MSN with n-[(3-trimethoxysilyl)propyl]ethy-lendiamine triacetic acid trisodium salt and with the cationic polymer ε-poly-l-lysine. The obtained positively charged DDS was loaded with rhodamine or vancomycin resulting in an antimicrobial nanodevice with high toxicity towards Gram negative *E.Coli* [50]. All those studies point out the importance of positively charged groups grafted on the external surface of MSNs.

The triethylenetetramine (TETA) molecule possess four amino groups that are easily protonated at physiological pH [51,52]. Rotello et al. showed that gold nanoparticles functionalised with TETA become positively charged and can efficiently load siRNA, serving as an antiviral DDS [53]. Unlike long-chain functionalisation moieties, which might clog MSN mesopores and thus negatively affect the drug adsorption and release, short-chained TETA might be a promising candidate as a positively charged functionalising agent for new DDS.

**Scheme 1:**
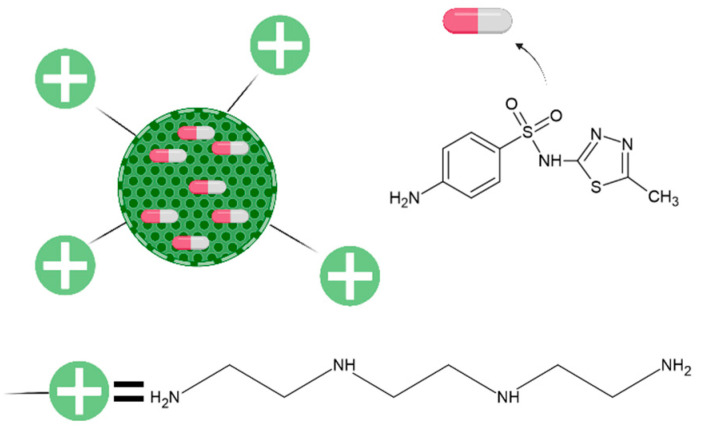
Schematic of MSN-TETA-SMZ as a possible antimicrobial nano-drug.

This work aims to prepare TETA functionalised-MSNs as carriers for the delivery of antimicrobial drugs (Scheme 1) [54]. To this purpose, a model drug, SMZ, was adsorbed on MSN-TETA and for comparison on bare MSNs. The samples were characterised through TEM, SAXS, FTIR, TGA, ELS and N_2_ adsorption/desorption isotherms. The kinetics and thermodynamics of SMZ adsorption were studied. Finally, the release of SMZ from MSN and MSN-TETA samples was investigated in a simulated body fluid (pH = 7.4, ionic strength = 150 mM) at T = 37 °C.

## 2. Results

### 2.1. MSN Structural Characterisation

**Figure 1:**
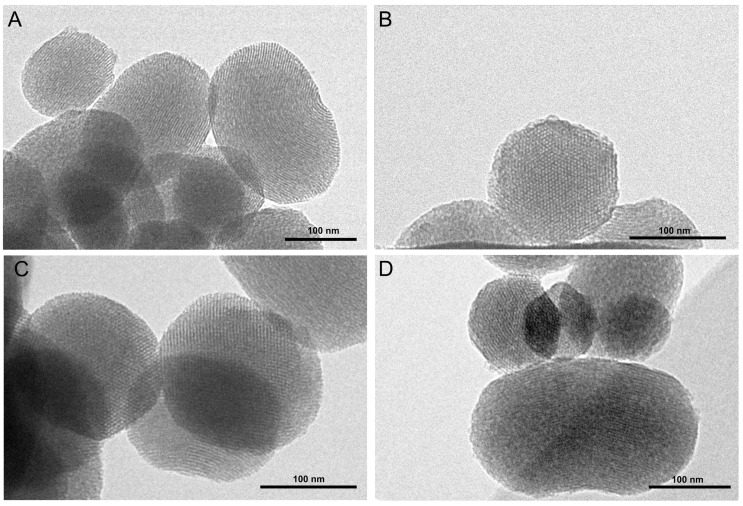
TEM images of MSN sample at different magnifications.

As described in Section 3.2, MSNs were synthesised using a CTAB template following a method applied in previous work [52]. Subsequently, a Cl-propyl moiety was introduced on the MSN surface to prepare MSN-Cl, which was further treated with TETA to obtain the functionalised MSN-TETA hybrid system. TEM images in Figure 1 show that most MSNs have a spherical or ellipsoidal shape with sizes around 100 nm. The cylindrical channels and the typical hexagonal array of pores (e.g., Figure 1B) can be seen for all porous nanoparticles.

**Figure 2:**
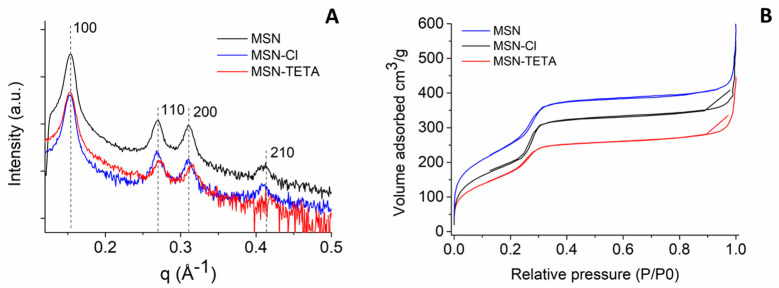
(**A**) SAXS patterns and (**B**) Adsorption/desorption N_2_ of MSN, MSN−Cl, MSN−TETA samples.

SAXS patterns of MSN, MSN-Cl and MSN-TETA samples showed a strong peak due to the (100) plane and other two weak peaks due to the (110) and (200) planes (Figure 2A). SAXS and TEM analyses confirm the characteristic MSN pattern (based on MCM-41 matrix), which corresponds to an ordered 2D hexagonal structure and p6mm space group [55]. The lattice parameter a is about 47 Å for MSN and the functionalised samples, confirming that the hexagonal array of pores is not affected by surface functionalisation.

Figure 2B shows the N_2_ adsorption/desorption isotherms of MSN, MSN-Cl and MSN-TETA samples. All curves show a sharp increase of N_2_ volume adsorption at a relative pressure P/P_0_ = 0.2–0.3, due to small-sized pores filled by relatively small volumes of N_2_. MSN had a BET surface area of 942 m^2^ g^−1^ that decreased after functionalisation, becoming 744 m^2^ g^−1^ and 671 m^2^ g^−1^ for MSN-Cl and MSN-TETA, respectively. The pore volume decreased consistently with TETA functionalisation (Table 1).

**Figure 3:**
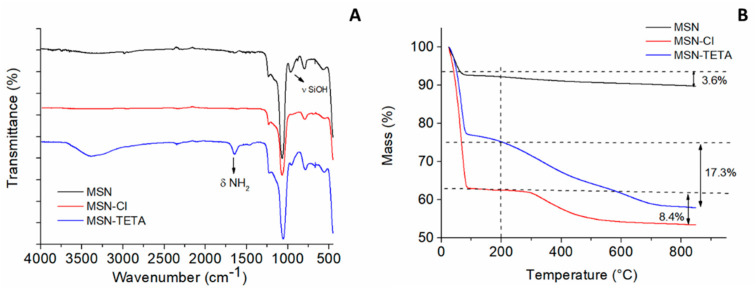
(**A**) FTIR spectra and (**B**) Thermogravimetric analysis of MSN, MSN−Cl and MSN−TETA.

The FTIR spectrum of MSN (Figure 3A) shows an intense broad peak at 1060 cm^−1^ and a less intense peak at 800 cm^−1^. These typical signals are assigned to the asymmetric and symmetric stretching vibrations of the Si-O-Si bond. Cl-propyl functionalisation is confirmed by the loss of the silanol peak at 976 cm^−1^, whereas MSN-TETA shows an additional peak at 1642 cm^−1^ which might be attributed to -NH_2_ bending.

The functionalisation of MSN samples was confirmed by thermogravimetric analysis (Figure 3B). Mass losses at T < 100 °C are attributed to the loss of humidity. Between 100 and 200 °C, no significant changes in mass are observed for all samples. However, above 200°C MSN-Cl loses a mass of 8.4% while MSN-TETA loses a mass of 17.3%. This difference (8.9%) is likely due to the TETA moiety. The functionalisation loadings calculated using TG analysis are listed in Table 1.

In addition, the functionalisation of MSN is also confirmed by zeta potential measurements carried out suspending the different samples in milli-Q water without background salt (Table 1). MSN sample has a negative zeta potential (ζ = −23 mV) due to the dissociation of the silanol groups at pH > 2–3 [56]. The sample MSN-Cl has a less negative zeta potential (ζ = −8 mV) likely since replacement of most silanols with Cl-PTES has occurred. MSN-TETA system displays a positive zeta potential (ζ = +27 mV) due to the functionalisation with triethylenetetramine groups, which are protonated in aqueous solution.

### 2.2. SMZ Adsorption on MSN and MSN-TETA

**Figure 4:**
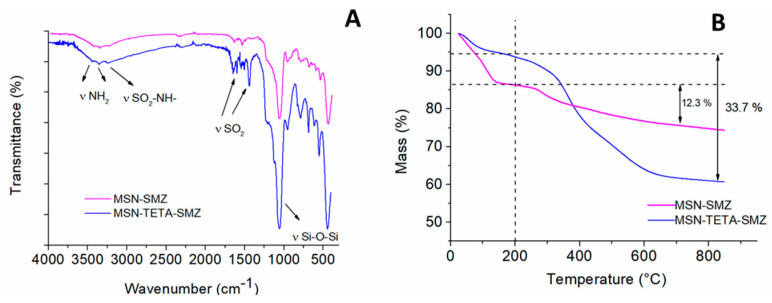
(**A**) FT-IR spectra (**B**) TGA of MSN-SMZ and MSN-TETA-SMZ.

SMZ drug was then adsorbed on MSN and MSN-TETA samples. SMZ presence on MSN is qualitatively confirmed by FTIR spectra (Figure 4A). The bands at 3447 e 3343 cm^−1^ are assigned to the symmetric and asymmetric stretching of aromatic -NH_2_ [57], while that at 3220 cm^−1^ is assigned to stretching of the S-N bond in SO_2_NH. The other two peaks, associated with O=S=O stretching (1450 e 1556 cm^−1^), and O=S=O bending (568 a 487 cm^−1^), confirm the loading of the drug on both MSN and MSN-TETA samples, respectively. A mass loss at T > 200 °C is observed for MSN-SMZ (12.3%) and MSN-TETA-SMZ (33.7%). Mass loss is entirely assigned to SMZ for MSN, while in the case of MSN-TETA, the loss of 33.7% can be only in part due to SMZ, since the decomposition of propyl-TETA moiety (17.3%) occurs in the same temperature range. These mass loss data, considered the contribution of the TETA moiety, correspond to a SMZ loading of 163 mg g^−1^ and 123 mg g^−1^ for MSN-TETA and MSN, respectively.

### 2.3. Adsorption Kinetics of Sulphamethizole on MCM-41 and MCM-41-TETA

**Figure 5:**
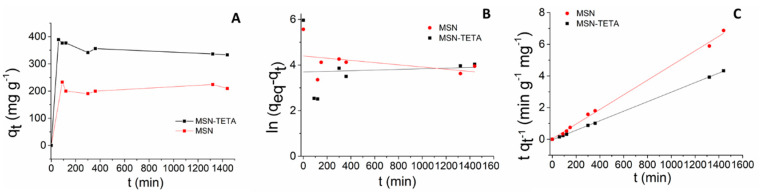
(**A**) Adsorption kinetics of SMZ on MSN and MSN-TETA fitted with (**B**) pseudo-first order and (**C**) pseudo-second order models.

The adsorption kinetics of SMZ on MSN and MSN-TETA was then studied. The adsorbed amount (*q_t_*, mg g^−1^) of SMZ drug as a function of the contact time is shown in Figure 5A. Both samples show a rapid *q_t_* increase within the first phase of adsorption reaching equilibrium after approximately 5 h. Data were analysed with pseudo-first-order (Figure 5B) and pseudo-second-order (Figure 5C) kinetic models [52,58]. The low R^2^ of the pseudo-first order model suggests that this model is not suitable to describe SMZ adsorption. On the contrary, the pseudo-second order model fits the experimental data quite well (R^2^ = 0.99 for both samples). This is also confirmed by the adsorbed amounts at equilibrium (*q*_eq_) of the experimental data compared against the data calculated by the pseudo-second order model. Indeed, calculated *q*_eq_ were 216.2 and 332.2 mg g^−1^ for MSN and MSN-TETA, respectively, with the experimental values being 232.9 and 389.1 mg g^−1^ for MSN and MSN-TETA, respectively (see Table 2). This finding agrees with Fukahori et al. who studied the adsorption kinetics of SMZ on zeolites at pH 3.8 [59].

### 2.4. Adsorption Isotherms of Sulphamethizole on MCM-41 and MCM-41-TETA

**Figure 6:**
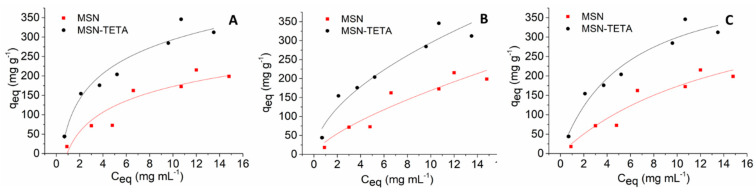
Adsorption isotherms (298 K) of SMZ on MSN and MSN-TETA fitted with Temkin (**A**), Freundlich (**B**), and Langmuir (**C**) models.

The adsorption isotherms of SMZ on MSN and MSN-TETA samples at 298 K were then determined. Figure 6 shows the SMZ adsorbed amount (*q_eq_*, mg_SMZ_/g_MSN/MSN-TETA_) vs. its equilibrium concentration in the adsorbing solution (*C_eq_*, mg_SMZ_/mL). The highest SMZ loadings were obtained with the functionalised MSN-TETA, corroborating the figures reported above from TGA (Figure 4). The experimental data were analysed using Freundlich, Temkin and Langmuir models as described in the experimental section [60,61].

Temkin (Figure 6A) and Freundlich (Figure 6B) models show a low correlation coefficient for MSN samples (R^2^ = 0.85 and R^2^ = 0.88, respectively). Langmuir (Figure 6C) model shows the best correlation coefficient for both MSN and MSN-TETA samples (R^2^ = 0.90 and 0.95, respectively) (Table 3). This suggests the formation of SMZ monolayer on both MSN and MSN-TETA, although a clear plateau is not reached within the explored concentration range. However, higher SMZ concentrations in the adsorbing solution could not be used because of solubility limitations. Jia et al. studied the adsorption behaviour of sulphadiazine (SDZ), sulphamethoxazole (SMX), and sulphadoxine (SDX) on a MIL-101(Cr)@GO metal organic framework [62]. They found a maximum loading capacity of 135.1 mg g^−1^ for SDZ, 101.0 mg g^−1^ for SMX and 119.1 mg g^−1^ for SDX. The estimated maximum monolayer coverage of SMZ found in our work is higher for MSN-TETA (465.3 mg g^−1^) than for MSN (442.0 mg g^−1^). In our experimental conditions, the highest adsorbed amount of SMZ is 345.8 mg g^−1^ for MSN-TETA and 215.4 mg g^−1^ for MSN samples (Figure 6). Interestingly, a significant higher loading is found for MSN-TETA system compared with MSN although functionalisation decreases the surface area available for SMZ adsorption, as demonstrated by N_2_-isotherms experiments. The higher SMZ adsorption obtained with MSN-TETA carrier might be due to the establishment of favourable interactions between the adsorbent and the adsorbate. Indeed, SMZ molecules are in anionic form already at pH > 5.3 (pI ~ 4 due to pKa_1_ = 2.1 ± 0.2 and pKa_2_ = 5.3 ± 0.2) [63]. It may be suggested that TETA groups, which make MSN-TETA surface positively charged, attract the negatively charged SMZ molecules, thus favouring a higher loading than that of bare MSN, as a result of electrostatic interactions. Similar behaviour was previously observed for ampicillin [64], sulphasalazine [65] and nucleic acids [66].

### 2.5. In Vitro Release of SMZ

**Figure 7:**
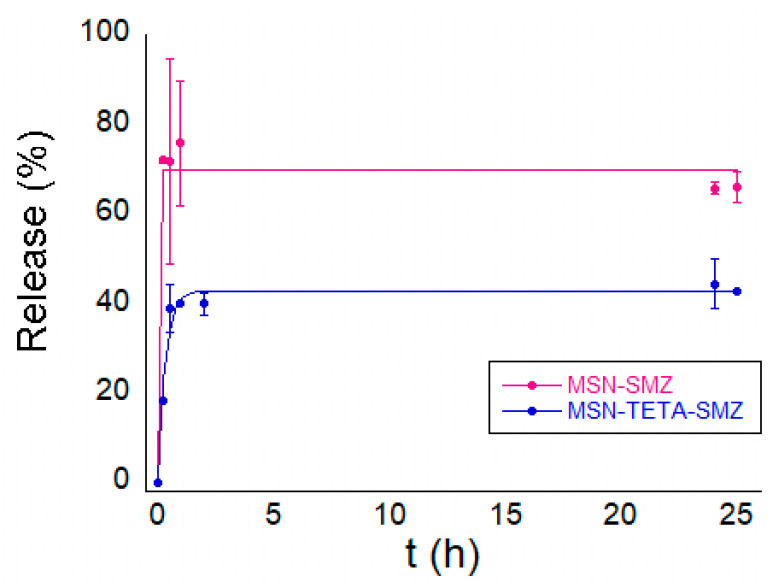
In vitro SMZ release of MSN and MSN-TETA.

In vitro release of SMZ from MSN and MSN-TETA carriers was then studied. The maximal released amount (A_max_%) of SMZ was 69.8% and 42.7% for MSN and MSN-TETA, respectively (Table 4). The kinetic release constant k_1_ has a very high value for bare MSN consistent with a burst release of the drug. In contrast, SMZ is released more slowly from MSN-TETA (k = 3.04 h^−1^), thus indicating that TETA functionalisation slows down drug release (Figure 7). The same attractive interactions that promote a higher loading of SMZ on MSN-TETA surface are likely responsible of the lower and slower drug release. The maximal amounts of released drug correspond to an SMZ concentration in the release solution of 171 µg mL^−1^ and 102 µg mL^−1^ for MSN and MSN-TETA, respectively. It is interesting to compare these concentrations with the dose of SMZ, or other similar drugs, in biological fluids. Sulfamethoxazole has a chemical structure similar to that of SMZ (an oxazole substitutes the thiadiazole moiety) and is also used in urinary tract infections as bacteriostatic in combination with trimethoprim [67]. After 8 h of oral administration, sulphamethoxazole reaches in an adult (24–34 years old) a blood concentration that varies from 98 to 128 µg mL^−1^ [68]. The active therapeutical sulfamethoxazole is counted in serum rather than in blood, with an optimal peak concentration of 100–150 µg/mL [69,70]. These values are comparable with the maximum SMZ concentration released by MSN and MSN-TETA found in the present work.

## 3. Materials and Methods

### 3.1. Materials

Hexadecyltrimethylammonium bromide CTAB (>99%), tetraethoxysilane TEOS (98%), anhydrous toluene (99.8%), triethylenetetramine TETA (≥97%), NaH_2_PO_4_ (99%), Na_2_HPO_4_ (99%), NaCl, NaOH, 3-chloropropyltrimethoxysilane CPTMS (≥97%), toluene, ammonium nitrate NH_4_NO_3_, sulphamethizole (99%) and dimethylformamide (DMF ≥ 99.9%), were purchased from Sigma Aldrich (Milano, Italy). Ethanol (99.8%) was provided by Honeywell.

### 3.2. Synthesis and Functionalisation of MSNs

MSN samples were synthesised as described elsewhere [64]. Briefly, 3.5 mL of NaOH (2 M) was added under stirring to 480 mL of H_2_O with 1 g of CTAB avoiding bubbles formation. The mixture was immersed in an oil bath at 80 °C with mild stirring. Before slowly adding 5 mL of TEOS with a syringe, stirring was increased up to 400 rpm. The reaction mixture was then kept at 80 °C for 2 h. The product was then collected, filtered and washed with water and ethanol. CTAB extraction was carried out by suspending 3 g of product in 1 L of a EtOH/H_2_O (95:5 *v*/*v*) solution containing NH_4_NO_3_ 10 g L^−1^, kept for 2 h at 80 °C. TETA functionalisation was conducted by a method similar to that reported in the literature for other silica materials [71]. Briefly, 0.7 mL of CPTMS was added to 1 g of MSN suspended in 25 mL of anhydrous toluene. The reaction mixture was left overnight under reflux at 110 °C. The obtained MSN-Cl was filtered, washed with toluene, ethanol and water and dried at 40 °C. A mass of 1.12 g of TETA were solubilised in 2 mL of DMF under heating for 1 or 2 min at ~30 °C. A mass of 1 g of MSN-Cl was then suspended in the TETA solution in DMF and left under stirring for 25 h at 110 °C.

### 3.3. Physico-Chemical Characterisations

MSN samples were characterised through transmission electron microscopy (TEM), small-angle X-rays scattering (SAXS), N_2_ adsorption–desorption isotherms, Fourier-transform infrared spectroscopy (FTIR), thermogravimetric analysis (TGA) and electrophoretic light scattering (ELS, zeta potential). TEM images were obtained on a JEOL 100S microscope. Small-angle X-ray scattering (SAXS) patterns were recorded with a S3-MICRO SWAXS camera system (HECUS X-ray Systems, Graz, Austria). The scattering patterns were recorded for 1 h. Textural analysis was carried out on an ASAP 2020 instrument, by determining the N_2_ adsorption/desorption isotherms at 77 K. Before analysis, MSN samples were heated at 250 °C at a rate of 1 °C/min under vacuum for 12 h. MSN-Cl, MSN-TETA and MSN-TETA-SMZ were heated at 25 °C at the rate of 1 °C/min under vacuum for 12 h. The Brunauer–Emmett–Teller (BET) [72] and Barrett–Joyner–Halenda (BJH) [73] methods were used to calculate surface area, pore volume and pore size distribution (from the desorption branch of N_2_ isotherm). FTIR studies were conducted with a Brüker Tensor 27 FTIR spectrometer equipped with a diamond-ATR accessory in the 4000–400 cm^−1^ range with a number of 128 scans at a resolution of 2 cm^−1^. TGA measurements were carried out in the T range 25–850 °C (heating rate = 10 °C/min), and under oxygen flow by means of a Perkin Elmer TGA7/DSC7.

### 3.4. Adsorption Kinetic Models

Kinetics of SMZ adsorption on MSN and MSN-TETA was determined [60] by suspending 10 mg of MSN or MSN-TETA in 1 mL of a SMZ solution (10 mg mL^−1^) in a mixture of EtOH/H_2_O 60:40, and left under rotation for 60, 100, 300, 360, 1320 and 1440 min at 25 °C. The supernatant was then assayed in a quartz cuvette taking 3 µL of solution and diluting it in 3 mL of a EtOH/H_2_O 60:40 solution. At different times, the supernatant of each sample was analysed through an Agilent Cary 60 UV–Vis spectrophotometer at 280 nm. The concentration of SMZ in the adsorption solution was calculated through a calibration curve (concentration range 0.5–20 mg mL^−1^). The residual SMZ concentration evaluated by UV–Vis and the corresponding adsorbed amount *q_t_* (mg g^−1^) was plotted vs. time. Kinetics measurements were analysed by different kinetic models named as:

Pseudo-first-order (differential form) [74]

(1) dqtdt=k′(qeq−qt)

for which the linearised form is expressed as:

(2) $$ln\left(q_{eq}-q_{t}\right)=lnq_{eq}-k\prime$$

*q_eq_* is the adsorbed quantity of adsorbate at the equilibrium and *q_t_* the adsorbed quantity (mg g^−1^) at time *t* (min), *k*′ is the pseudo first order kinetic constant expressed in min^−1^.

Alternatively, the pseudo-second-order model was used [75]:

(3) dqtdt=k′(qeq−qt)

for which the linearised form is expressed as:

(4) $$\frac{t}{q_{t}}=\frac{1}{\left(k\prime \prime q_{eq}{}^{2}\right)}+\frac{t}{q_{eq}}$$

where *k*″ is the pseudo-second-order kinetic constant (g mg^−1^ min^−1^).

### 3.5. Adsorption Isotherms

Adsorption isotherm studies were carried out by suspending 10 mg of MSN or MSN-TETA in 1 mL of a solution of SMZ dissolved in a EtOH/H_2_O 60:40 mixture at different concentrations (from 1.1 to 16.7 mg mL^−1^) under constant rotation for 24 h at 25 °C. The SMZ equilibrium concentration in the supernatant, obtained by centrifugation for 2 min at 1500 rpm, was then assayed by UV–Vis spectroscopy. A volume of 3 µL of the supernatant was diluted in 3 mL of a solution EtOH/H_2_O 60:40 in a quartz cuvette. The absorbance of SMZ solutions was measured through an Agilent Cary 60 UV–Vis spectrophotometer at 280 nm, and the concentration of SMZ in the adsorption solution was calculated with the appropriate calibration curve (concentration range 0.5–20 mg mL^−1^). Adsorption data were fitted through Freundlich, Temkin, and Langmuir models.

Freundlich model describes the adsorption isotherm as [76]:

(5) $$q_{eq}=K_{F}C_{eq}^{1/n}$$

In which, *q*_eq_ is the adsorbed amount (mg of adsorbate/g of adsorbent), *C_eq_* (mg mL^−1^) is the equilibrium concentration, *K_F_* (support capacity, L mg^−1^) is Freundlich constant and *n* (heterogeneity factor) is a dimensionless parameter that describes the heterogeneous adsorbent surface. Both empirical parameters are specific for adsorbent–adsorbate interactions at a specific temperature. With heterogeneous adsorption sites, a formation of multilayer is to be expected.

The Langmuir model [77]:

(6) $$q_{eq}=\frac{q_{max}K_{L}C_{eq}}{1+K_{L}C_{eq}}$$

where, *q_eq_* is the adsorbed amount at the equilibrium (mg g^−1^), *q_max_* is the maximum cover capacity (mg g^−1^), *K_L_* is Langmuir constant (L mg^−1^) and *C_eq_* (mg mL^−1^) is the equilibrium concentration. Langmuir model assumes that the adsorbent is homogeneous, and all the sites are equivalent, and only a monolayer of molecules is permitted.

Temkin model [78]:

(7) $$q_{eq}=\frac{RT}{b_{T}}\ln\left(A_{t}C_{eq}\right)$$

where, *b_T_* is the Temkin constant (J mol^−1^), *R* is ideal gas constant (J mol^−1^ K^−1^), *T* is the absolute temperature (K), *A_T_* is the isotherm equilibrium binding constant (mL mg^−1^) and *C_eq_* (mg mL^−1^) is the equilibrium concentration. According to Temkin model, the indirect effect of adsorbent–adsorbate interactions matters. It assumes that the adsorption enthalpy of adsorbed drug is due to an increase in surface covering.

### 3.6. Kinetics of SMZ Release

SMZ release kinetic studies were carried out suspending 50 mg of MSN-SMZ or MSN-TETA-SMZ samples in different vessels containing 50 mL of 100 mM phosphate buffer pH 7.4 NaCl 150 mM (PBS) at 37 °C. To analyse the SMZ release, other withdrawals were made at different times within 25 h. Each withdrawal was made of 2 mL, and the same amount of liquid was reconstituted with PBS buffer in each vessel to maintain “sink conditions” [79]. Results were analysed with an appropriate calibration curve and reported in percentage by the equation [80]:

(8) $$\frac{M_{t}}{M_{0}}\left(\%\right)=A_{max}\left(1-e^{-kt}\right)$$

where *M_t_* (mg) is drug mass at time of the withdrawal *t*, *M*_0_ (mg) is the mass of drug loaded, *A_max_* is the maximum drug release and *k*_1_ (h^−1^) is the release constant rate.

## 4. Conclusions

Herein, a new DDS based on SMZ loaded on MSN and MSN-TETA is reported. TEM and SAXS structural characterisations confirmed the successful synthesis of MSN having the typical hexagonal structure. N_2_-isotherms of MSN showed a surface area of 942 m^2^ g^−1^ that, as expected, and decreased because of material functionalisation. TGA and FTIR techniques were used to characterise functionalised and post-adsorption samples, highlighting a higher drug loading with MSN-TETA (up to 345 mg g^−1^), despite its lower surface area value (671 m^2^ g^−1^), with respect to that of MSN (up to 215.4 mg g^−1^). SMZ adsorption kinetics on both MSN and MSN-TETA was consistent with a pseudo-second-order model, whereas the adsorption isotherms were consistent with the Langmuir model. The SMZ release of both materials was studied achieving up to 69.8% (171 µg mL^−1^) for MSN and 42.7% for MSN-TETA (102 µg mL^−1^). The latter concentration is very similar to the concentration of similar drugs measured in the blood. Interestingly, SMZ release was slower from MSN-TETA (*k*_1_ = 3.04 h^−1^) compared with MSN (*k*_1_ = 136 h^−1^). These findings highlight the importance of the establishment of suitable drug–carrier interactions. Indeed, as observed here, the positive charged functionalisation with TETA moieties may favour the interactions with the negatively charged SMZ rather than the negatively charged MSN surface. The attractive electrostatic interaction between SMZ and MSN-TETA surface is responsible for a higher loading and a slower release compared with the SMZ-MSN pair. The present DDS, based on SMZ loaded on MSN-TETA obtained here, could be potentially used against Gram negative bacteria such as *E. coli* to treat urinary tract infections. The therapeutic antibacterial activity of the promising DDS will deserve to be tested in a future work.

## Figures and Tables

**Table 1:** Surface area (S_BET_), pore volume (V_p_), lattice spacing (*a*), zeta potential (ζ) and functionalization loadings (L) of MSN, MSN-Cl and MSN-TETA samples.

Sample	^a^ S_BET_ (m^2^g^−1^)	^b^ V_p_ (cm^3^g^−1^)	^c^ dp (Å)	^d^*a* (Å)	^e^ ζ (mV)	^f^ L (µmol g^−1^)
MSN	942	0.78	25	48	−23	-
MSN-Cl	744	0.77	25	47	−8	770
MSN-TETA	671	0.62	24	46	+27	469

^a^ Specific surface area calculated by the BET equation. ^b^ Pore volume from the desorption branch calculated at P/P0 = 0.99 by BJH method. ^c^ Pore diameter calculated by BJH method using desorption branch isotherm. ^d^ Lattice parameter obtained by SAXS patterns. ^e^ Zeta potential obtained in suspending particles in distilled water. ^f^ Functionalisation of propyl-Cl and propyl-TETA loadings calculated by TGA.

**Table 2:** Kinetic parameters for SMZ adsorption on MSN and MSN-TETA.

		Pseudo-First Order			Pseudo-Second Order		
Sample	*q_eq_*_exp_ (mg g^−1^)	k’ (min^−1^)	*q_eq_*_calc_ (mg g^−1^)	R^2^	k” (g mg^−1^ min^−1^)	*q_eq_*_calc_ (mg g^−1^)	R^2^
MSN-SMZ	232.9	4.8 × 10^−4^	81.3	0.17	6.4 × 10^−4^	216.2	0.99
MSN-TETA-SMZ	389.1	1.4 × 10^−4^	40.2	0.005	2.9 × 10^−4^	332.2	0.99

**Table 3:** Adsorption isotherm parameters for SMZ on MSN and MSN-TETA.

Sample		Temkin		Freundlich				Langmuir		
	q_exp_ (mg g^−1^)	b_t_ (J mol^−1^)	A_T_ (mL mg^−1^)	R^2^	K_F_ (L mg^−1^)	n	R^2^	K_L_ (L mg^−1^)	q_max_ (mg g^−1^)	R^2^
MSN-SMZ	215.4	37.5	1.1	0.85	33.5	1.4	0.88	0.08	442.0	0.90
MSN-TETA-SMZ	345.8	25.7	2.1	0.95	83.7	1.8	0.94	0.18	465.3	0.95

**Table 4:** Kinetic parameters for SMZ release from MSN and MSN-TETA.

Samples	k_1_ (h^−1^)	A_max_ (%)	A_max_ (µg mL^−1^)	R^2^
MSN-SMZ	136	69.8	171	0.98
MSN-TETA-SMZ	3.04	42.7	102	0.96

## Data Availability

Not applicable.
